# Measuring the Equilibrium Spreading Pressure—A Tale of Three Amphiphiles

**DOI:** 10.3390/molecules29174004

**Published:** 2024-08-24

**Authors:** Boyan Peychev, Dimitrinka Arabadzhieva, Ivan L. Minkov, Iglika M. Dimitrova, Elena Mileva, Stoyan K. Smoukov, Radomir I. Slavchov

**Affiliations:** 1 School of Engineering and Materials Science, Queen Mary University of London, Mile End Road, London E1 4NS, UKs.smoukov@qmul.ac.uk (S.K.S.); 2 Rostislaw Kaischew Institute of Physical Chemistry, Bulgarian Academy of Sciences, Acad. G. Bonchev Str., bl. 11, 1113 Sofia, Bulgaria; dimi@ipc.bas.bg (D.A.); minkov.ivan@gmail.com (I.L.M.); imd@uctm.edu (I.M.D.); mileva@ipc.bas.bg (E.M.); 3 Department of Chemistry, Biochemistry, Physiology, and Pathophysiology, Faculty of Medicine, Sofia University, 1 Koziak Str., 1407 Sofia, Bulgaria; 4 Department of Physical Chemistry, Faculty of Chemical Technologies, University of Chemical Technology and Metallurgy, 8 Kl. Ohridski Blvd., 1756 Sofia, Bulgaria

**Keywords:** solid amphiphiles, monolayers, equilibrium spreading pressure, surface spreading, surface dissolution, experimental protocol

## Abstract

A surfactant’s equilibrium spreading pressure (ESP) is the maximum decrease in surface tension achievable at equilibrium below the Krafft point. Difficulties in measuring the ESP have been noted previously but no well-established experimental protocols to overcome them exist. We present a case study of three solid amphiphiles with different propensities to spread on the air–water interface. Starting with the partially water soluble n-dodecanol (C_12_H_25_OH), which spreads instantaneously. The strong Marangoni flows associated with the spreading result in the dislocating of the Wilhelmy plate or crystals attaching to it. A temporary mechanical barrier in front of the spreading crystals mitigates the flows disturbing the plate. Presaturating the subphase with the amphiphile prevents the establishment of dynamic steady states, reduces the standard error by a factor of three and causes faster equilibration. The perfluoroalkylated analog of dodecanol (11:1 fluorotelomer alcohol, C_11_F_23_CH_2_OH) is slow spreading. With surfactant crystals on the interface, the surface pressure reaches a pre-equilibrium plateau within an hour, followed by equilibration on day-long timescales. We show that it is better to estimate the ESP by averaging the values of multiple pre-equilibrium plateaus rather than waiting for equilibrium to be established. Finally, the nonspreading amphiphile DPPC exhibits a large barrier for the mass transfer from the DPPC crystal to the aqueous surface. This was overcome by introducing a volatile, water-immiscible solvent deposited on the surface next to the crystals to facilitate the spreading process and leave behind a monolayer.

## 1. Introduction

When an insoluble surfactant, in the form of surfactant crystals or droplets, is brought into contact with an aqueous surface, it spreads—a process of a “surface dissolution” of the amphiphile takes place to produce a mono- or multilayer [1]. The surfactant layer in equilibrium with the surfactant bulk phase is called the equilibrium spread layer. The respective drop in surface tension is called the equilibrium spreading pressure (ESP) and is an important characteristic of the surfactant. The ESP is the maximum attainable equilibrium surface pressure and sets a limit on the monolayer stability [2] below the Krafft point. Much like the CMC, the ESP is also a surface saturation limit, making it an important parameter where applications of surfactants are considered (especially water-insoluble ones). For example, when making synthetic lung surfactants, the ESP of the components has to be considered to ensure the spontaneous formation of a dense monolayer in an expanded lung [3]. Furthermore, the ESP marks a reference point where the chemical potential of the monolayer is independent of the substrate on which it is spread [4,5]. Thus, the ESP of a surfactant as a function of the type and concentration of electrolytes in the subphase has proven useful in a recently develop methodology for measuring the adsorption of ions on monolayer-covered surfaces [5,6,7]. In our recent work, we have been studying equilibrium spread monolayers on the surface of aqueous solutions of electrolytes of medium to high concentrations (0.5–3 M), as they allow for the determination of the electrolyte adsorption.

Unfortunately, the ESP has proven to be difficult to measure, especially for solid amphiphiles. One way to determine the ESP of an amphiphile is as the maximum pressure of a compression isotherm [4,8], provided that the measured isotherm corresponds to equilibrium rather than a metastable supercritical monolayer. As the monolayer is compressed, right above the ESP, a new 3D phase starts to form. This corresponds to a plateau on the equilibrium compression isotherm. The formation of 3D structures is broadly referred to as the “collapse” of the monolayer. However, if the monolayer is compressed too rapidly, it can be overcompressed to pressures well above the ESP. At a certain point of overcompression, the monolayer goes through a plastic failure or fracture, which shows up either as a plateau or a spike on the compression isotherm [8]. In such cases, the beginning of the 3D nucleation process appears as a slight, often difficult to notice, increase of the monolayer compressibility [8], before the plateau or spike where the plastic failure or fracture occurs. Furthermore, the fracture of a monolayer in a dynamic isotherm resembles a monolayer at equilibrium with a 3D phase in an equilibrium isotherm, both of which are often reported as monolayer collapse. Therefore, the term “monolayer collapse” is somewhat ambiguous.

As long as the rate of compression is slow enough to avoid dynamic effects, determining the ESP as the equilibrium collapse pressure of the monolayer can be relatively easy for surfactants that are liquid in their bulk form [8]. However, often that might require intermittent compression, i.e., waiting for the surface pressure to settle between compression steps to avoid overcompression. For surfactants that are solid in their bulk phase, the monolayer relaxation processes are much slower and, thus, avoiding overcompression is much harder even with intermittent compression. Furthermore, most modern setups are designed to compress continuously at constant speeds. This can often lead to significant deviations from the equilibrium behaviour near the collapse point.

Since obtaining the ESP from a compression isotherm can be problematic, the ESP is usually determined by putting a surfactant bulk phase in direct contact with the surface and observing the decrease in the surface tension that corresponds to the spreading of the surfactant. For liquid surfactants, one deposits oil lenses on the surface with a pipette or a glass rod. For solid surfactants, there are two common approaches—either powder is deposited on a clean surface or a suspension is made and transferred to the measuring container [9]. Measuring the ESP of liquid surfactants is easier and carries less experimental uncertainty than the measurement of the ESP of solid surfactants [10]. This is because measuring the ESP of solid surfactants is often complicated by a number of additional issues. Some typical complications that may arise include the following:

(i) The common method for obtaining the spreading pressure is through meniscus weight measurement (Wilhemly plate, Du Nouy ring). The measuring probe, e.g., a Wilhelmy plate, can be contaminated, which skews the results. The main reason for probe contamination, in these systems, is that solid bodies attract each other through capillary interactions at the surface [11]. Given enough time for the amphiphile crystals to spread over the whole surface, those that pass close enough to the probe will stick to it. Furthermore, the solvent evaporation at the three-phase contact line may lead to surfactant build-up on the probe via the coffee-ring effect [12]. The immediate concern with probe contamination is that the added weight of the crystals changes the measurement readout, since the method relies on weight measuring. However, the amphiphile deposited on the probe also changes the shape and perimeter of the three-phase contact line. Furthermore, contact between the probe and the solid may change the contact angle of the probe itself through, e.g., multilayer deposition on its surface. In our experience, the wetting behaviour of a Wilhelmy platinum plate is visibly different before and after a spreading pressure experiment. Since commercial systems assume a constant probe weight and perimeter of the three-phase contact line, any change would introduce an error into the surface pressure value printed on the screen. The variation in these parameters is possible to account for numerically, but is not straightforward (see, e.g., [13]). Instead, these errors can be minimised by shortening the length of the experiment to reduce the deposition on the probe.

(ii) The crystal dissolution and the relaxation of the dense monolayer may exhibit slow kinetics [14,15,16]. As much time as several days might be needed to achieve a constant spreading pressure value [1,9,17]. Such long timescales carry risk for the contamination of the surface, the chemical degradation of the surfactant (due to, e.g., autooxidation and hydrolysis), and, as discussed already, deposit buildup on the measuring probe. Furthermore, solvent evaporation may be significant at these timescales. The common Langmuir troughs are shallow to ensure a large surface area-to-volume ratio. Thus, the water from a standard trough might evaporate completely within a 24 h period. In a meniscus-measuring method, the goal is to position the probe as close to the level of the interface as possible in order to avoid additional buoyancy forces acting on it. From a practical point of view, usually one would use a differential approach, i.e., directly measuring the surface pressure (the change in meniscus weight) instead of the surface tension (meniscus weight), in which case, the buoyancy forces cancel out, as long as the liquid level is constant. A change in the water level would leave an uncompensated force acting on the measuring probe and would introduce an error in the measurement (see the Supplementary Materials). The problem is partially suppressed by the tendency of surfactant monolayers to inhibit evaporation [18,19]. Minimising the effect from evaporation can be achieved in several ways: by reducing the working temperature, by saturating the surrounding air with the solvent vapours or by minimising the time of the experiments. Whether these are sufficient to justify the length of the experiment depends on the system and experimental time in question.

(iii) Another source of uncertainty when working with solid surfactants is the possibility of co-existing polymorphic crystalline phases [15,20] of different chemical potentials. The crystalline particle size distribution can alter the measurements in a similar manner [21], via the Kelvin effect, i.e., different sized particles have different chemical potentials. Furthermore, the surface defects on the crystals (e.g., the number of sharp edges) may similarly cause the increased activity of the surfactant. Due to these effects, a single sample of a solid surfactant phase may hold particles of varying chemical potentials, leading to the value of the measured spreading pressure being dynamic rather than in equilibrium.

(iv) There could be an uptake of solvent into the surfactant phase. This would result in a slightly different chemical potential compared to the pure surfactant phase, which is reflected by a different spreading pressure. For solid surfactants, the swelling is generally a slow process. However, in a bulk suspension equilibrated overnight, the surfactant phase is more hydrated than it would be when dry crystals are deposited on the surface. Thus, reported values for the spreading pressure might differ depending on whether they refer to a dry or a solvent-saturated solid surfactant [17]. For liquid surfactants, the problem is further exacerbated, as the dissolution of the solvent into the surfactant can occur at a similar timescale to that of the spreading process.

(v) A quasi-steady state of the spread monolayer can be established if the surfactant is of non-negligible solubility [22]. In such a case, the rate of dissolution of the monolayer to the aqueous phase balances the rate of the surface dissolution of the bulk amphiphile (the surfactant crystals) towards the monolayer. In a quasi-steady state, the surface pressure is nearly constant even though the ESP is not reached.

(vi) Because the relaxation processes in a dense monolayer can be slow, there might be kinetically stabilised metastable states established [3,9].

Perhaps due to the listed complications, finding well-defined experimental protocols for measuring the spreading pressure of a given system in the literature is difficult. In fact, most of the literature on the subject of ESP measurement techniques is from before 1990 and somewhat outdated. In this article, we present a case study of three different solid amphiphiles spreading on aqueous surfaces, each presenting a different challenge, and the ways we found most appropriate to determine their apparent spreading pressures. The focus is on evaluating the approaches to overcome some common challenges in measuring the ESP. We consider substrates of increased salinity, which resemble the typical conditions for the application of surfactants in drilling fluids [23,24], and which are important for our method for measuring electrolyte adsorption on monolayers [5,6,7].

## 2. Results and Discussion

### 2.1. Water Solubility and Probe Contamination—n-Dodecanol

The first amphiphile we are going to be looking at is n-dodecanol. The solid form of dodecanol is a white polycrystalline substance. It generally comes packaged in a plastic bottle as a solid. However, dodecanol melts at around room temperature (approx. 24 °C), which means that in ambient conditions, it is a mix of both solid and liquid (often in a paste-like form). To avoid the uncertainty with regards to its exact phase composition, in this work, we focused on measuring the ESP of solid dodecanol at 10 °C.

When dodecanol is deposited onto an aqueous surface, it readily spreads and lowers the surface tension almost instantly. Within 10 min, the surface pressure settles at a fairly constant value (≤0.1 mN/m change per 10 min), which we take to be the apparent spreading pressure. One issue that needs to be addressed is the solubility of the dodecanol. Dodecanol is sparingly soluble; its solubility in neat water is ∼5 × 10^−6^ M at 10 °C [25]. This means that, until the subphase is saturated, there is a constant mass transfer away from the surface towards the bulk of the subphase, which can offset the results. To avoid this mass transfer, one can presaturate the subphase with the studied substance. Typically, we would dispense dodecanol crystals in a flask with the desired subphase composition (either neat water or electrolyte solutions) and leave it to equilibrate overnight at the working temperature (10 °C).

To illustrate the advantages of presaturating the subphase, Table 1 presents a comparison of the apparent spreading pressures we determined by spreading n-dodecanol on neat water and on a presaturated aqueous dodecanol solution. As can be seen, the pre-saturation has only a small effect on the absolute value of the measurement. In fact, the difference between the results with and without presaturation is comparable to the method accuracy (0.1–0.2 mN/m). Likely, in this specific case, the solubility of the alcohol is low enough and the spreading process is fast enough that the dissolution process has a small effect on the apparent spreading pressure. However, from Table 1 it is clear that, when working with a presaturated aqueous dodecanol solution, the results are more reproducible [6]. The standard error of the mean is almost three times higher in the case of neat water. Furthermore, the surface pressure tends to settle faster on the presaturated solution.

The likely reason for the lower value of the apparent spreading pressure on unsaturated water and the higher spread of the data is the formation of quasi-steady states near the equilibrium. During the spreading of a soluble amphiphile on the surface, there are several processes taking place simultaneously:

(i) At the air–crystal–water three-phase contact line, alcohol molecules are detaching from the crystals and spreading on the air–water interface [1,26].
(ii) Alcohol molecules from the monolayer are submerging into the subsurface layer [25].
(iii) Alcohol molecules from the subsurface layer are diffusing into the subphase, proportional to the undersaturation of the solution.

As the monolayer density approaches the density of the monolayer at equilibrium with the bulk surfactant phase, the rate of surface dissolution (process (i)) decreases, while the rate of bulk dissolution (process (iii)) increases. Thus, a quasi-steady state is established, where the mass transfer from the crystals to the surface is balanced by the mass transfer from the surface towards the bulk solution. Under such conditions, the monolayer density and the surface pressure will evolve slowly. Of course, given enough time, if there is an excess of surfactant deposited on the surface, it will saturate an undersaturated solution [27], i.e., if our experiments are extended, the results on an unsaturated subphase should match the results on the presaturated subphase. However, both the longer experimental times and the inherent ambiguity of the question of “is the surface pressure constant enough to be considered constant” can be avoided altogether by presaturating the solution with the alcohol.

Another problem, common to most amphiphiles, that needs to be addressed, is the attachment of crystals to the probe. One option to minimise the attachment of particulates to the probe is to deposit only a small amount of crystals. From our experience, for all systems, 10 mg of amphiphile crystals is generally sufficient for the experiment. A smaller sample (<10 mg) may be sufficient, but it is hard to measure and transfer it to the surface in a reproducible manner. For the amphiphiles in this article, these 10 mg are distributed over tens to hundreds of particles, and only occasionally a few of those stick to the probe. For our system, 4 mg of weight added to the probe corresponds to approximately 1 mN/m of change in the measured surface pressure, i.e., the added weight problem is lessened by the small amount of deposited surfactant. By comparing the weight of a wet plate at the beginning of the experiment to the weight of the plate at the end of the experiment with particles attached to it, we find that the added error of the increased weight is of the order of 0.2 mN/m (assuming all stuck particles remain on the plate when it is lifted), which is acceptable. However, the magnitude of error due to the change in the three-phase contact line is more difficult to evaluate.

**Figure 1:**
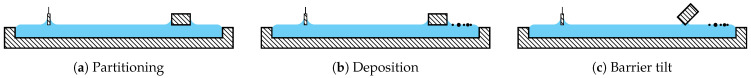
A schematic representation of the procedure for determining the apparent spreading pressure of dodecanol. After depositing the crystals behind the barrier, the barrier is carefully tilted around its edge before completely removing it.

A problem specific to this system is the high rate of surface spreading. Typically, one would deposit the crystalline powder far away from the probe. During the experiment, the particles slowly spread over the surface through Brownian motion, slowed down by the dense monolayer. However, in the case of dodecanol, since the surface solubility/rate of spreading is so high, the initial contact of the crystal with the surface causes a strong Marangoni flow that spreads the powder towards the probe immediately. In fact, this flow is so strong that, without precautions, it can slant the Wilhelmy plate with respect to the surface—the initial pulse pushes the plate to the side and the resultant dense solid monolayer impedes the relaxation of the probe to its equilibrium position. To avoid this undesired effects, we have devised the following procedure [6] (see Figure 1):

(i) The system is set up and the balance is zeroed.
(ii) The surface is partitioned in two with a barrier.
(iii) The crystals (approx. 10 mg) are added to the side opposite the measuring probe.
(iv) After several minutes have passed for the surfactant to spread, the barrier is carefully tilted on its edge. This allows the alcohol to leak slowly behind the barrier.
(v) Eventually, the barrier is fully removed to allow for unobstructed spreading.

When done correctly, the procedure leaves the crystalline powder grouped in one end of the trough and prevents the initial shock to the Wilhelmy plate. The dense monolayer that is formed impedes the movement of the particulates, thus, lowering the amount of probe contamination. While the crystals are not perfectly immobilised, we find the results to be fully satisfactory.

### 2.2. Slow Kinetics of Spreading—11:1 Fluorotelomer Alcohol

The second amphiphile we are going to be looking at in this article is 11:1 fluorotelomer alcohol (CF_3_(CF_2_)_10_CH_2_OH). It is a structural analog to the aliphatic dodecanol, having the same chain length and polar headgroup, but a fluorocarbon tail instead of a hydrocarbon one. This alcohol is an off-white polycrystalline flaky solid, with a melting temperature around 110 °C (according to the vendor). Furthermore, it has practically no solubility in water, as can be expected from an intermediate-length PFAS. The fluorine has not only a profound effect on the melting temperature and the solubility of the alcohol, but on its spreading behaviour as well; the fluorotelomer alcohol CF_3_(CF_2_)_10_CH_2_OH is an example of a surfactant exhibiting very slow kinetics of spreading.

**Figure 2:**
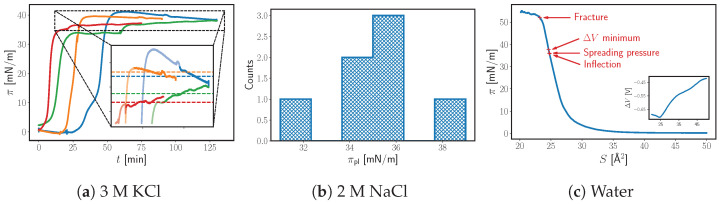
(**a**) The surface pressure of CF_3_(CF_2_)_10_CH_2_OH deposited on 3 M of a KCl solution as a function of time at 10 °C. The dashed lines present an averaged surface pressure of the plateaus $\pi_{\mathrm{pl}}$. (**b**) A histogram of the surface pressure of the plateaus $\pi_{\mathrm{pl}}$ observed when CF_3_(CF_2_)_10_CH_2_OH spreads on 2 M of a NaCl solution (count = 7, 10 °C). (**c**) The dynamic compression isotherm of CF_3_(CF_2_)_10_CH_2_OH on neat water at 10 °C and with a 2.5 mm/min rate of compression.

In a typical experimental procedure, after the system is set up and the balance is zeroed, we would deposit, far away from the measuring probe, approx. 10 mg of fluoroalcohol crystals on the surface of the subphase. We found that the procedure we used with the fatty dodecanol to limit the initial spread with the barrier was unnecessary here. The spreading of the fluoroalcohol was slow enough that the Marangoni flow was not an issue. Presaturation of the subphase was also unnecessary, as the fluoroalcohol has negligible solubility, but we conducted it anyway to ensure that the results for the two alcohols can be compared. After deposition on the surface, the surface pressure π reaches a “pre-equilibrium” plateau value $\pi_{\mathrm{pl}}$ (see Figure 2a), usually within an hour, and then continues to creep slowly (approx. 1 mN/m per hour), presumably towards the equilibrium. In our experiments, we did not observe a truly constant value within 5.5 h, which is unreasonably long for our specific setup (following the reasoning presented in the Introduction).

Notice that, after reaching the pre-equilibrium plateau, in the final stages of equilibration, π sometimes increases and sometimes decreases (Figure 2a). Furthermore, during the slow relaxation period (after an hour in Figure 2a), we have not observed any maxima or minima that cannot be classified as noise, i.e., the final relaxation is overall monotonous. Therefore, the plateau pressures $\pi_{\mathrm{pl}}$ should give us an interval within which the ESP ($\pi_{\mathrm{sp}}$) lays (see Figure 2a). Furthermore, the plateau pressures $\pi_{\mathrm{pl}}$ seem to be normally distributed (see Figure 2b). We assume that the mean of $\pi_{\mathrm{pl}}$ and $\pi_{\mathrm{sp}}$ coincide. Thus, instead of tackling the many complications associated with longer run times, we collected pre-equilibrium surface pressures from multiple shorter runs and then averaged them for an estimate of the apparent ESP.

At present, there is no formula for how long the runs should be. On the one hand, the time for reaching pre-equilibrium varies between repetitions (e.g., Figure 2a); sometimes it would take less than 20 min, sometimes more than an hour. Fortunately, this does not seem to correlate with the surface pressure of the plateaus. Thus, there is a stochastic element to the run time necessary for the experiment. On the other hand, while there is no clear effect of the composition of the subphase on the “lag time”, the reproducibility of the surface pressure of the pre-equilibria plateaus is statistically different between systems. As can be seen in Table 2, adding an electrolyte scatters the data (higher standard deviation). The effect is ion-specific, e.g., NaBr has a much bigger effect on the standard deviation of the results than NaCl. The effect of the electrolyte on the reprudicibility of the data suggests that the ions play some role in the spreading process, which is not surprising as there is clear evidence of specific interactions between ions and uncharged monolayers [5,7]. Often, for the systems with higher standard deviation, we had to carry out on average longer experiments to offset the difference (see Table 2).

This approach of determining the spreading pressure, through averaging the plateau values from multiple short runs, should lead to higher deviations, i.e., less precise measurement. For instance, in Table 2, a comparison is given between the shorter and the longer runs for CF_3_(CF_2_)_10_CH_2_OH spreading on 3 M of a NaBr solution (the least reproducible system we came across). If we take the subset of only the longest experiments (3.5 to 5.5 h), the standard deviation is lower than that of the shorter experiments (less than 3.5 h), as one would expect. The difference between the two sample means seems large (∼2.5 mN/m); however, they are within one standard deviation of each other, i.e., this is not a statistically significant difference. Furthermore, as it was discussed in the introduction, we expect there to be hard-to-evaluate sources of error associated with longer experiments (e.g., contamination, evaporation, etc.). Thus, extending the experiment beyond a certain point should lower the accuracy of the measurement, i.e., there is a tradeoff between precision and accuracy. We find that, for CF_3_(CF_2_)_10_CH_2_OH, a good compromise is to keep the experiment shorter than 4 h. This is enough time for pre-equilibrium to be established (see Figure 2a), giving us a good estimate of the ESP.

To check whether the averaging method gives meaningful results, we also measured compression isotherms, focusing on pure water as a subphase (Figure 2c). The isotherms are in qualitative agreement with the results of Takiue and Vollhardt [28]. In our π-*S* isotherms, the surface pressure reaches values as high as 52 mN/m before fracture, well above the apparent spreading pressure we determined or expected, i.e., the monolayer before fracture is overcompressed. This is to be expected, since the slow kinetics of the spreading process correspond to a slow reverse process of nucleation in the monolayer. When the alcohol is spreading on the surface, in the final stages of relaxation, the surface pressure changes at ∼1 mN/m per hour. In the case of the compression isotherm, even the slowest speed of compression the software allows cannot match such slow processes, i.e continuous compression cannot allow for equilibration near the ESP. Thus, the plateau above 52 mN/m must correspond to a fractured monolayer, not one equilibrated to the normal surfactant phase. By interpolating (π as function of *S* as a 5-th degree spline) the region of the isotherm where the surface pressure grows rapidly (>5 mN/m) but is before the fracture, we find an inflection point that is just below the apparent spreading pressure (see Table 3). The inflection point (increase of the monolayer compressibility) should correspond to the beginning of the 3D nucleation and growth processes, i.e., it should occur around the ESP. We repeated the experiment at several speeds and found that compressions with ≤5 mm/min give visibly similar isotherms, whereas 10 mm/min or higher leads to a large shift in the isotherm. However, the surface pressures at which the inflection occurs do increase appreciably from 2.5 mm/min to 5 mm/min (see Figure 2c), as a result of overcompression. A rate of compression of 2.5 mm/min is sufficiently slow and gives an inflection within one standard deviation of the apparent spreading pressure (see Figure 2c).

It should be kept in mind that such an inflection might also represent a different physical process, e.g., second-order phase transition, the onset of surfactant dissolution, etc. Therefore, we also measured the change in the contact potential difference due to the monolayer ΔV (inset on Figure 2c). The ΔV potential is used to study monolayers as it is proportional to the total normal dipole moment of the surface, i.e., proportional to the average orientation of the amphiphile molecules and the monolayer density. As can be seen, ΔV decreases upon compression, corresponding to an increase in the total surface dipole moment (by absolute value), until 24.6 Å^2^ per molecule (at 2.5 mm/min compression), where ΔV starts to increase again. This behaviour would suggest the formation of lamellar structures parallel to the surface, in which the normal dipole moments of the opposing molecules cancel out, i.e., a 3D phase starts to form. In comparison, the hard-disk area of the fluoroalcohol should be approx. 27 Å^2^ [29], i.e., the 24.6 Å^2^ per molecule area corresponds to an overcomppressed monolayer that is starting to collapse. The extremum of ΔV appears at a surface pressure just above the apparent spreading pressure that was determined (see Figure 2c and Table 3).

Overall, the coincidence between the isotherm inflection, the ΔV extremum, and the apparent spreading pressure leads us to conclude that the approach and assumptions made are valid for this particular system, i.e., the determined apparent spreading pressure is close to the ESP. This motivates us to tentatively view the averaging method as preferable to day-long runs in cases where slow kinetics of equilibration with the bulk amphiphile are observed. We find that this approach offers a good balance between uncertainty due to stopping the experiment too soon and far from equilibrium, and avoiding the uncertainty associated with long experiments (contamination, deposition of particulates on the probe, and evaporation).

### 2.3. Nonspreading—Dipalmitoylphosphatidylcholine

The final substance we will discuss is dipalmitoylphosphatidylcholine (DPPC), a biologically important amphiphile. Physically, it is a white, low-density crystalline powder. DPPC undergoes a phase transition near human body temperature ($T_{m}∼41$°C [30]). Below its “melting” temperature, DPPC bilayers are in their “gel” phase which is highly ordered [31], while above that temperature, they melt to a “liquid-crystalline” state.

In general, phospholipids do not readily spread on the aqueous surface below their gel-to-liquid crystal melting temperature [3,8,20,30,32]. Our observations showed that, below that temperature, upon dispensing DPPC particles (in their gel phase) on the surface of water, barely visible oil lenses form around the particulates. This may be an indication of limited spreading and the formation of multilamellar structures on the surface. However, the reading for the surface tension barely changes (≤0.2 mN/m). On the other hand, if a monolayer is made from a DPPC solution, it can be compressed up to quite high surface pressures without collapsing (up to 70 mN/m [3,8,30,33,34]). Specifically, with intermittent compression, the DPPC monolayer is stable up to ∼45 mN/m [30]. This can only be explained as a kinetic stability of either the monolayer or the solid in contact with an aqueous surface. It appears that the material exchange between the 3D phase and the monolayer is hindered by a very high energy barrier of spreading [3], which we refer to as “nonspreading”.

One proposed explanation of the nonspreading of lipids is that, for spreading to occur, water has to hydrate them, i.e., the swelling of the particulates has to occur beforehand [20]. However, this cannot happen below the gel-to-liquid-crystalline transition temperature [20], i.e., DPPC in its gel form does not swell. On the other hand, it has been found that, above $T_{m}$, the ESP measured from dry lipids is the same as that measured from a suspension, but is settles faster [9,30]. This may suggest that, in fact, the hydration impedes, rather than promotes, the spreading process, possibly due to a change in the wetting behaviour of the powder [35].

Assuming that the system is indeed kinetically stable, we need a way to lower the barrier for the DPPC molecules to separate. Since DPPC dissolves in some organic solvents, one would suppose that adding such a solvent to the system would reduce the energy barrier and may allow the DPPC molecules to cover the water/solvent interface. The solvent would then evaporate leaving a DPPC monolayer on the water’s surface. Assuming the evaporation of the solvent is slow compared to the other processes happening at the surface, it should be possible to reach the equilibrium between the 3D lipid phase and the final monolayer. Thus, we devised the following procedure [7]:

(i) The system is set up and the balance is zeroed.
(ii) Approximately 10 mg of DPPC crystals are deposited on the surface far away from the Wilhelmy plate.
(iii) After a short period, ca. 30 μL of chloroform is added evenly onto the surface, with a Hamilton syringe, in a drop-wise manner. This step is repeated every 10 min.

**Figure 3:**
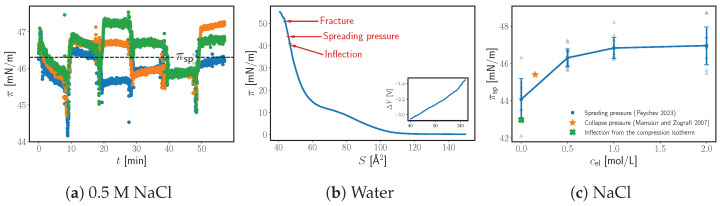
(**a**) The surface pressure of DPPC deposited onto 0.5 M of NaCl at 25 °C as a function of time for three different experiments. The origin t=0 corresponds to the entire initial dose of chloroform being deposited onto the surface (1–2 min after the first drop of chloroform is added). The jumps at 10 min intervals follow subsequent additions of chloroform. The dashed line represents the unweighted average of the plateau values (apparent spreading pressure). (**b**) The dynamic compression isotherm of DPPC on neat water at 25 °C and with a 2.5 mm/min rate of compression. (**c**) The apparent spreading pressure of DPPC as a function of NaCl concentration in the subphase at 25 °C. The semi-transparent triangles show the spreading pressure at each plateau from multiple runs. The error bars are the standard deviation. For comparison, a collapse pressure from the data in the literature is added (star), as well as the inflection pressure from the compression isotherm (x) [7,30].

Figure 3a shows three distinct runs of a typical experiment in a 0.5 M NaCl solution. Before the chloroform, the surface pressure does not change within the experimental uncertainty. As soon as we start adding the chloroform, the surface pressure rises immediately and significantly. By the time the entire syringe is deposited the surface pressure is already above 40 mN/m. As can be seen, at t=0 (all the chloroform is added), there is a significant relaxation process that lasts for more than 10 min (enough for the chloroform to evaporate). Therefore, we would keep adding more portions of chloroform at 10 min intervals. After the third portion onwards, the surface pressure usually settles quickly to a constant value (≤0.1 mN/m of change in 10 min). Not only does this suggest that the monolayer is close to an equilibrium, since the surface pressure is practically constant, but that the chloroform has no discernible effect on the equilibrium, since the constant surface pressure is often reached almost immediately while the chloroform should take several minutes to evaporate.

The plateau values are reproducible between runs (see Figure 3a) and exhibit no specific pattern of appearance. The large variance in the plateaus could be intrinsic to the monolayer at equilibrium with the bulk amphiphile itself (e.g., due to an evolving size, shape, and position of the particulates as a result of the addition of chloroform). Alternatively, it could be that, once again, we have stable pre-equilibria that relax very slowly even in the presence of the organic solvent. The exact processes that are occurring and the mechanism of the monolayer formation warrant further investigation in a dedicated future study. We nevertheless assume that, to estimate the ESP, it is sufficient to perform the experiment as described and average the plateau surface pressures.

To validate our results, we look again at the compression isotherm (Figure 3b). At a 2.5 mm/min speed of compression, there is an inflection point (found as before) just below the spreading pressure (see Figure 3c). This inflection point is reproducible within, e.g., ±3 mN/m, which is a much larger uncertainty compared to the fluorotelomer alcohol. Unlike the fluorotelomer alcohol, the ΔV curve of DPPC exhibits no extremum. This likely indicates a different mechanism of collapse, e.g., the formation of bilayer folds that are perpendicular to the surface [36] rather than parallel. However, to further examine the validity of our results, we compared them with the collapse pressures at intermittent compression. Mansour and Zografi have measured those for DPPC in 0.15 M of a NaCl solution at 25 °C as well as the spreading pressure of DPPC at 45 °C [30]. The two coincide very well, since, as they have shown, the spreading pressure of the choline lipids is weakly dependent on the temperature [30]. Figure 3c shows the effect of the concentration of NaCl on the apparent spreading pressure of DPPC from our investigation [7]. As can be seen, our results and Mansour and Zografi’s intermittent collapse pressure seem to lay on the same curve. The good agreement between these two sets of results, as well as the compression isotherm inflection, suggests that our approach produces physically meaningful results.

### 2.4. Summary

In this paper, we address the methodology of determining the ESP of solid amphiphiles. We highlight possible complications to the experiment, some of which are universal and some of which are substance-dependent; in the course of our recent research, every surfactant we came across presented a different experimental challenge and required a different approach.

For dodecanol, there are two main concerns when measuring its spreading pressure—the solubility and the tendency of crystals to stick to the measuring probe, driven by a strong Marangoni flow. We show that presaturating the subphase can prevent complications and errors connected to the dissolution of the surfactant. Furthermore, we have developed a mechanical method of limiting the rate of surface mass-transfer in order to delay the contamination of the measurement probe.

For the 11:1 fluorotelomer alcohol, the major complication is the very slow kinetics of equilibration. When the amphiphile is deposited onto the surface, usually within an hour, the surface pressure reaches a pre-equilibrium where the rate at which the monolayer evolves is very slow, on day-long timescales. We argue that, when determining the spreading pressure, such long experimental timescales can be avoided by averaging the values of the pre-equilibria plateaus. That is based on the simple observation that the final equilibrium value seems to be approached both from above and from below. Averaging out these initial values is demonstrated to give a reasonable estimate of the ESP. While this approach has a lower precision than waiting until no change is observable, it removes experimental uncertainties related to long runs, leading to higher accuracy, i.e., the optimal time per run is one that is sufficient for the initial plateau to be established but not much longer.

Finally, DPPC does not spread on the aqueous surface at room temperature the way other amphiphiles do. The mass transfer from the bulk to the aqueous surface seems to be hindered by too large of an activation energy. We show that adding chloroform can facilitate the spreading process. In this way, we were able to measure the apparent spreading pressure of DPPC at room temperature, previously thought to be unachievable. A comparison with the data in the literature for the collapse at intermittent compression and with the inflection of a dynamic compression isotherm shows that the spreading pressure we measured is indeed close to the ESP. The exact mechanism of the solvent-aided spreading is an interesting prospect for future research.

## 3. Methods and Materials

### 3.1. Materials

Prior to use, the NaCl (≥99.8%, Sigma-Aldrich: St. Louis, MO, USA), KCl (≥99.5%, Fluka: Buchs, Switzerland), and NaBr (≥99%, Acros Organics: Geel, Belgium) were calcinated to remove any organic impurities. The temperature was increased gradually for 15 min to 400 °C and then the sample was left to cool down. Chloroform (Sigma-Aldrich), dipalmitoylphosphatidylcholine (DPPC, Avanti Polar lipids: Alabaster, AL, USA), dodecanol (CH_3_(CH_2_)_10_CH_2_OH, ≥98 %, Sigma Aldrich) and 11:1 fluorotelomer alcohol (CF_3_(CF_2_)_10_CH_2_OH, Fluorochem: Hadfield, UK) were used without further purification. All solutions were prepared volumetrically with double-distilled water (GFL 2001/2 distiller).

### 3.2. Spreading Pressure

The spreading pressure was measured with a commercial KSV Nima (Biolin Scientific, Gothenburg, Sweden) surface balance with a platinum Wilhelmy plate. The whole system was enclosed in an acrylic box. A PTFE trough with dimensions of 195.0 × 50.0 mm was used. The ambient temperature was controlled with an air-conditioning unit. The subphase temperature was kept constant with a precise thermostat (Eco Silver RE415, LAUDA DR. R. WOBSER GMBH & CO. KG, Lauda-Königshofen, Germany). Before each experiment, the Wilhelmy plate and glassware were cleaned with a chromic acid solution and rinsed thoroughly with distilled water. The trough was cleaned repeatedly with ethyl alcohol and chloroform until a value for the surface tension of water was achieved that was in line with that in the literature.

### 3.3. Compression Isotherms

For the compression isotherms, a bigger PTFE trough with dimensions of 580 × 145 mm was used. The change in the contact potential difference was measured with an additional vibrating plate capacitor module attachment (KSV SPOT, Biolin Scientific, Gothenburg, Sweden). In a typical experiment, 50–70 μL of a chloroform surfactant solution (approx. 1 g/L) was deposited onto the surface in a drop-wise manner using a Hamilton syringe. After ca. 10 min for the solvent to evaporate, a constant-speed compression was started. The compression was symmetrical, with both barriers moving at the same speed.

## Figures and Tables

**Table 1:** The apparent spreading pressure of n-dodecanol on water at 10 °C from multiple measurements, with an without the presaturation of the subphase.

	Measurements [mN/m]								Mean [mN/m]	Standard Error [mN/m]
Unsaturated	46.57	47.11	46.87	46.85	46.06	46.37	46.43	46.52	46.60	0.11
Presaturated	46.84	46.81	46.68	46.66	-	-	-	-	46.75	0.04

**Table 2:** The apparent spreading pressure of CF_3_(CF_2_)_10_CH_2_OH on different systems at 10 °C. The experimental time specifies the minimum and maximum duration of an experiment. The sample size is the number of experimental runs carried out.

System	Experimental Time	Sample Size	Mean [mN/m]	Standard Deviation [mN/m]	Standard Error [mN/m]
Water	<3.5 h	4	36.08	0.34	0.17
3 M NaCl	<2 h	5	42.37	0.83	0.37
3 M KCl	<2.5 h	4	41.11	1.02	0.51
	<5.5 h	6	38.02	3.23	1.32
3 M NaBr	<3.5 h	3	36.72	3.22	1.86
	3.5h<…<5.5 h	3	39.31	2.68	1.55

**Table 3:** Comparison between the determined apparent EPS of CF_3_(CF_2_)_10_CH_2_OH on neat water and the surface pressures at which the CF_3_(CF_2_)_10_CH_2_OH compression isotherms have an inflection and at which the corresponding ΔV goes through a minimum, at two different rates of compression and 10 °C.

Apparent Spreading Pressure = 36.08 mN/m				
**Standard Deviation = 0.34 mN/m**				
	**Inflection**		ΔV **Minimum**	
**Rate**	**Surface Pressure [mN/m]**	**Area per Molecule [Å^2^]**	**Surface Pressure [mN/m]**	**Area per Molecule [Å^2^]**
2.5 mm/min	35.82	24.80	37.64	24.63
5.0 mm/min	38.77	24.97	38.90	24.97

## Data Availability

The dataset is available upon request from the authors.

## Supplementary Materials

The following supporting information can be downloaded at: https://www.mdpi.com/article/10.3390/molecules29174004/s1, A discussion on the effect of the evaporation of water on the apparent ESP.
