# Supplementary material for: Measuring the Equilibrium Spreading Pressure—A Tale of Three Amphiphiles
Source: Molecules. 2024 Aug 24;29(17):4004. doi: 10.3390/molecules29174004 (PMC11396376; doi:10.3390/molecules29174004)
Supplement: Supplementary file 1 [file molecules-29-04004-s001.zip › molecules-3150795-supplementary.pdf]

Supplementary Materials: Measuring the Equilibrium Spreading Pressure — a Tale of Three Amphiphiles

Boyan Peychev[1,2], Dimitrinka Arabadzhieva[2], Ivan L. Minkov[2,3], Iglika M. Dimitrova[2,4], Elena Mileva[2], Stoyan K. Smoukov[1] and Radomir I. Slavchov[1,*]

S1. Water evaporation

When measuring the surface tension gravimetrically, the goal is to position the measuring probe on level with the fluid. That way only the pull of the meniscus is measured. If the probe is partially submerged below the surface, due to the displaced water, there is an additional buoyancy force acting on the probe. This leads to an experimental error in the order of ~ 0.2 mN/m. Often, to avoid this error one would measure the change of the weight of the probe after it is brought in contact with the surface, i.e. the surface pressure rather than the surface tension. That way the buoyancy force cancels out. However, for long experiments the level of the fluid may change due to evaporation or condensation. This leaves an uncompensated buoyancy force skewing the results.

Let us evaluate this bias in a worst case scenario — the highest temperature we are working with (25 °C), no surfactant to reduce the evaporation rate, dry air, and a waft (air flow). At 25 °C and 46.4 mL/min dry air flow, the evaporation rate constant of water is $3.26 * 10^{-7}$ mol/s cm^2 [1]. This corresponds to a loss of

$$\text{(evaporation rate constant)} * \text{(molar mass)} = 0.02 \text{ g/h cm}^2 \tag{S1}$$

of water, leading to a lowering of the water level by

$$\text{(loss of mass per hour per surface area)} * \text{(density)} = 0.02 \text{ cm/h}. \tag{S2}$$

The volume of water displaced by the Wilhelmy plate is proportional to the thickness of the plate, the width of the plate, and the immersion. Therefore, the displaced water changes by

$$\text{(thickness)} * \text{(width)} * \text{(change of water level)} = 0.4\ \mu\text{L/h}. \tag{S3}$$

As we said in the article, for our setup 4 mg of the weight acting on the plate equals 1 mN/m change of the surface tension. Thus, the error introduced by the evaporation of the water is 0.1 mN/m per hour. This is an acceptable bias when compared with the intrinsic uncertainty of the ESP measurements (see "Results and Discussion" in the main article).

Another source of error to consider is the rising electrolyte concentration. For low electrolyte concentration it is negligible. However, in the extreme case of 3 M electrolyte, 0.02 g/h cm^2 loss of water corresponds to the electrolyte concentration increasing in the order of $\sim$ 4% per hour (assuming 50 mL solution volume in a through with 100 cm^2 surface area). Furthermore, it is important to note that, even at these high evaporation rates, no direct effect from the evaporation on the structure of the monolayer should be expected.

All of this gives us an upper limit to the experimental error we might expect from the evaporation of the solvent. For our experiments, in all cases, the setup is enclosed in an acrylic box which reduces the air flow; there is a dense monolayer which may impede the evaporation substantially [1]; the ambient humidity is different from zero, and in some cases the temperature is lower. As a result, the actual experimental error due to the evaporation must be considerably lower (at least by an order of magnitude). Thus, we are confident that, for up to 5 hour experiments, it is an acceptable error, while for daylong experiments the humidity needs to be controlled more precisely.

References

1. Rusdi, M.; Moroi, Y. Study on water evaporation through 1-alkanol monolayers by the thermogravimetry method. *Journal of colloid and interface science* **2004**, *272*, 472–479.

Disclaimer/Publisher's Note: The statements, opinions and data contained in all publications are solely those of the individual author(s) and contributor(s) and not of MDPI and/or the editor(s). MDPI and/or the editor(s) disclaim responsibility for any injury to people or property resulting from any ideas, methods, instructions or products referred to in the content.
